# Impact of violacein from *Chromobacterium violaceum* on the mammalian gut microbiome

**DOI:** 10.1371/journal.pone.0203748

**Published:** 2018-09-13

**Authors:** Heidi Pauer, Cristiane Cassiolato Pires Hardoim, Felipe Lopes Teixeira, Karla Rodrigues Miranda, Davi da Silva Barbirato, Denise Pires de Carvalho, Luis Caetano Martha Antunes, Álvaro Augusto da Costa Leitão, Leandro Araujo Lobo, Regina Maria Cavalcanti Pilotto Domingues

**Affiliations:** 1 Laboratório de Biologia de Anaeróbios, Departamento de Microbiologia Médica, Instituto de Microbiologia Paulo de Góes, Universidade Federal do Rio de Janeiro, Rio de Janeiro, RJ, Brazil; 2 Escola Nacional de Saúde Pública Sergio Arouca, Fundação Oswaldo Cruz, Rio de Janeiro, RJ, Brazil; 3 Laboratório de Interação Hospedeiro-Microbiota, Instituto de Biociências, Universidade Estadual Paulista, Campus do Litoral Paulista, São Vicente, SP, Brazil; 4 Faculdade de Farmácia, Universidade Federal do Rio de Janeiro, Campus Macaé, Macaé, RJ, Brazil; 5 Faculdades Integradas Aparício Carvalho, Porto Velho, RO, Brazil; 6 Instituto de Biofísica Carlos Chagas Filho, Universidade Federal do Rio de Janeiro–Rio de Janeiro, Brazil; 7 Instituto Nacional de Ciência e Tecnologia em Inovação em Doenças de Populações Negligenciadas, Centro de Desenvolvimento Tecnológico em Saúde, Fundação Oswaldo Cruz, Rio de Janeiro, RJ, Brazil; East Carolina University Brody School of Medicine, UNITED STATES

## Abstract

Violacein is a violet pigment produced by *Chromobacterium violaceum* that possesses several functions such as antibacterial, antiviral, antifungal, and antioxidant activities. The search for potential compounds and therapies that may interfere with and modulate the gut microbial consortia without causing severe damage and increased resistance is important for the treatment of inflammatory, allergic, and metabolic diseases. The aim of the present work was to evaluate the ability of violacein to change microbial patterns in the mammalian gut by favoring certain groups over the others in order to be used as a therapy for diseases associated with changes in the intestinal microflora. To do this, we used male Wistar rats, and administered violacein orally, in low (50 μg/ml) and high (500 μg/ml) doses for a month. Initially, the changes in the microbial diversity were observed by DGGE analyses that showed that the violacein significantly affects the gut microbiota of the rats. Pyrosequencing of 16S rDNA was then employed using a 454 GS Titanium platform, and the results demonstrated that higher taxonomic richness was observed with the low violacein treatment group, followed by the control group and high violacein treatment group. Modulation of the microbiota at the class level was observed in the low violacein dose, where Bacilli and Clostridia (Firmicutes) were found as dominant. For the high violacein dose, Bacilli followed by Clostridia and Actinobacteria were present as the major components. Further analyses are crucial for a better understanding of how violacein affects the gut microbiome and whether this change would be beneficial to the host, providing a framework for the development of alternative treatment strategies for intestinal diseases using this compound.

## Introduction

Violacein is a violet pigment produced as a secondary metabolite by several Gram-negative bacteria, including *Chromobacterium violaceum* [1], *Janthionobacterium lividum* [2], *Alteromonas luteoviolacea*, *Pseudoalteromonas luteoviolacea* and *Duganella* sp. *B2* [3]. Its biosynthesis begins with L-tryptophan, and is catalyzed by enzymes VioA, VioB, VioE, VioD, and VioC, successively, which are encoded by the *vioABCDE* operon [4]. Secondary metabolites often have functions other than just being byproducts of the metabolic processes of bacteria during growth and propagation. These molecules can be biologically active and give a competitive edge against antagonistic species. Thus, pharmacological properties showed by many of these secondary metabolites have demonstrated a potential use of these molecules in clinical practice [5]. Violacein has been associated with various biological properties such as a potential cancer therapeutic activity due to cytotoxic effects against several tumor cell lines. This compound shows cytotoxicity at IC_50_ values that mainly range in the submicromolar concentrations [5], with an apoptosis effect on HL60 leukemic cells [6], inhibition of Akt-mediated signal transduction in human colon cancer cells [7], and growth inhibition against Ehrlich ascites tumor [8].

Violacein also presents antibacterial activity against *Bacillus licheniformis*, *Bacillus subtilis*, *Bacillus megaterium*, *Staphylococcus aureus*, *Mycobacterium tuberculosis*, *Pseudomonas aeruginosa*, and others [1,9–11]. The mechanism involved in violacein antibacterial activity remains unclear. However, it has been suggested that this molecule can affect cell viability by decomposing components essential for life maintenance inside the cell, since this violet pigment leads to cell death, not only inhibition of bacterial growth [3]. Also, the antibacterial activity of violacein varies significantly depending on the microorganism tested [12].

Natural pigments, like violacein, displays lower toxicity and higher decomposition rate than synthetic pigments and are, thus, often used as food preservatives [11,13]. Violacein also has activity against nanoflagellates [14], *Leishmania* sp. [15], and *Trypanosoma cruzi* [16]. Activity against herpes simplex virus and polioviruses after infection of HeLa cells has also been described [17]. Additionally, anti-ulcerogenic activity has also been reported [18]. Other interesting activities that have been observed include potent immunomodulatory, analgesic and antipyretic effects [19], as well as antioxidant activity [20]. Violacein is a hydrophilic molecule, which means it can efficiently pass through the cell membrane. A protective effect against UV radiation has also been suggested for this pigment [21,22].

Violacein is produced naturally by various bacterial species, including, as mentioned above, the saprophyte bacterium *C*. *violaceum*, normally considered nonpathogenic to humans. Nevertheless, this species can occasionally act as an opportunistic pathogen in animals and humans, and may cause fatal septicemia from skin lesions, with many liver and lung abscesses [23]. *C*. *violaceum* is a free-living Gram-negative bacillus and facultative anaerobe found in soil and water samples of tropical and subtropical areas of several continents [1]. *C*. *violaceum* is abundant in the black waters and banks of Negro river in the Brazilian Amazon region [24]. Riverine populations living along the banks of Amazonian Rivers use its water for consumption [25].

The human gastrointestinal tract houses a complex microbial ecosystem, the gut microbiota. This intestinal ecosystem is partially responsible for maintaining human health by acting as a barrier against invasion of pathogens and contributing to important metabolic functions [26]. Many metagenomics studies have revealed the association of imbalances in gut microbiota populations with inflammatory, allergic, and metabolic diseases, such as inflammatory bowel disease (IBD), diabetes, autism, obesity and asthma [27–32]. The intestinal microbiota shows marked stability and constancy, but can be altered by endogenous and exogenous factors, including diet, antimicrobials and stresses [33]. The search for potential therapies and substances that may interfere and modulate these microbial consortia without causing severe and increased drug resistance is important for treatment of these dysbioses. Thus, we evaluated the ability of violacein to change microbial patterns in the mammalian gut microbiome. Our results indicate that violacein consumption affects the intestinal microbiome, and provide a framework for further studies aimed at investigating the potential of this compound as a therapy for diseases associated with changes in the intestinal microbiota.

## Materials and methods

### Animals and housing conditions

Two-month-old male Wistar albino rats were randomly divided into three groups. Group A and B (n = 6 per group) received, respectively, 50 μg/mL and 500 μg/mL of violacein daily and group C (n = 4) received only vehicle (drinking water with DMSO). Rats received 100 μL violacein directly in the mouth (half-dose twice a day) by gavage for a month. It is important to point out that the dose of violacein was kept constant throughout the experiment. Therefore, the ratio between the amount of violacein per animal weight unit decreased over the duration of the experiment due to animal weight gain. Animals were housed (18 cm x 31 cm x 38cm) with hardwood-shaving bedding and (two cages per group) with a 12-hour light/dark cycle at 25±1°C at a relative humidity of 60–70%, and had access to standard chow and water *ad libitum*. Health of animals (weight, consistency of stool, blood in stool, polyuria and polydipsia) was monitored every day before gavage and no relevant characteristic was observed. All experiments were performed in the animal laboratory. At the end of the treatment, animals were humanely euthanized by decapitation, and whole intestinal content was collected for DNA extraction. All animal experiments were conducted according to the ethical guidelines of and approved by the Ethics Committee on Animal Use (*Comissão de Ética no Uso de Animais*—*CEUA—*097/16) from *Universidade Federal do Rio de Janeiro (CCS-UFRJ)*.

### Violacein extraction

*C*. *violaceum* was cultivated aerobically at 30°C in Luria-Bertani (LB) medium and the growth inoculated in LB agar for violacein extraction. The amount of 50 g of *C*. *violaceum* collected from the plates was added to 1 L of acetone and shaken for 30 min. Then, the cells were filtered with Whatman^®^ qualitative filter paper, Grade 1 (Sigma, China). The filter was washed with 500 mL of acetone, shaken for 30 min and filtered again. The solution was kept at room temperature until 80% of the acetone evaporated. In the remaining solution, 37% hydrochloric acid was gradually added until a blue/green color was obtained and then two volumes of sterile distilled water were added. After 48 h, the solution was centrifuged at 12000 x *g* for 10 min. The supernatant was discarded and the pellet washed three times with sterile distilled water and then dissolved in ethanol. After 24 h ethanol was fully evaporated to obtain the violacein crystals [34]. The violacein crystals were solubilized in water containing DMSO 5%.

### DNA extraction from intestinal content

DNA was isolated from approximately 200 mg of whole intestinal content using two different commercially-available kits: QIAamp DNA Stool Minikit (Qiagen, Hilden, Germany) and MoBio PowerSoil DNA Isolation Kit (MoBio Laboratories, Carlsbad, USA). The first step of DNA extraction was performed with the QIAamp kit, with the lysis incubation at 95°C for 5 minutes, instead of 70°C. The lysate was transferred to the PowerBead Tubes included in the MoBio kit and the next steps were performed according to the manufacturer's instructions. DNA was stored at -20°C until analysis.

### PCR amplification for Denaturing Gradient Gel Electrophoresis

16S rRNA genes were amplified using universal bacterial primers PRBA338fGC (5'-CGC CCG CCG CGC GCG GCG GGC GGG GCG GGG GCA CGG GGG GAC TCC TAC GGG AGG CAG CAG-3') and PRUN518r (5'-ATT ACC GCG GCT GCT GG-3') targeting the V3 region (230 bp) [35]. Each reaction had a total volume of 50 mL and contained 10x PCR buffer (Invitrogen, São Paulo, Brazil), 50 mM MgSO_4_ (Invitrogen, São Paulo, Brazil), 200 μM of each deoxynucleoside triphosphate (Promega, Madison, USA), 50 μM of each primer, 0.5 U of Platinum *Taq* DNA polymerase (Invitrogen, São Paulo, Brazil) and 50 ng of DNA as template. PCR conditions were 92°C for 2 min, followed by 35 cycles of denaturation at 92°C for 1 min, annealing at 55°C for 30 s and extension at 72°C for 1 min and then a final extension step at 72°C for 6 min. Amplification was performed in a Veriti^®^ 96-Well Fast Thermal Cycler (Applied Biosystems, Singapure). Amplification products were verified by gel electrophoresis before proceeding to Denaturing Gradient Gel Electrophoresis (DGGE) analysis.

### Analysis of intestinal microbiota by Denaturing Gradient Gel Electrophoresis

DGGE was performed using a DCode system universal mutation detection system (Bio-Rad, Richmond, USA). The amplicons were applied directly to the gel containing 8% (w/v) polyacrylamide and 0.5 x TAE (20 mM Tris-acetate [pH 7.4], 10 mM sodium acetate, 0.5 mM EDTA) with a gradient of 50–65% denaturant (urea and formamide). The electrophoresis run was for 16 h at 60°C and 75 V. After electrophoresis, the gel was stained for 30 min with SYBR Green (Molecular Probes, Oregon, USA) and scanned with STORM™ 860 Imaging System (GE Healthcare, Milwaukee, USA). The cluster analysis and Pearson correlation coefficients (r) were performed by the unweighted pair group method with average linkages (UPGMA) using BioNumerics Software (Applied Maths, Belgium). The DGGE band profiles were also converted into data matrices using the Bionumerics v6.0 package. To analyze the differences between profiles and composition of bacterial communities, matrices were ordered by non-metric dimensional scaling (NMDS) [36,37] using a Bray–Curtis distance matrix with Past 3.x Software [38]. To assess the variation between different samples (A, B and Control group), a permutational multivariate analysis of variance (PERMANOVA) [39] was performed using Past 3.x Software [38].

### Preparation of samples for pyrosequencing

A barcoded pyrosequencing approach was employed for in-depth analysis of the bacterial community composition and diversity. To this end, we selected representative animals from each group, two animals from the control group as well as three animals each from the low and high violacein dose groups were used. A thorough description of the pyrosequencing sample preparation is provided in S1 Protocol. Briefly, the V1-V3 and V3-V5 hypervariable regions of the bacterial 16S rRNA gene were PCR-amplified with the primer pairs 27F (5’-AGA GTT TGA TCC TGG CTC AG-3’)-534R (5’-ATT ACC GCG GCT GCT GG-3’) and 357F (5’-CCT ACG GGA GGC AGC AG-3’)-926R (5’-CCG TCA ATT CMT TTR AGT-3’), respectively, according to the “Human Microbiome Project (HMP) 454 16S Protocol Version 4.2.2″ (S1 Protocol). The protocol is available on the HMP Data Analysis and Coordination Center website (http://www.hmpdacc.org/). These PCRs generated amplicons of approximately 500 and 560 bp in length for V1-V3 and V3-V5 regions, respectively. The samples were sequenced following the aforementioned protocol. Amplification primer pairs were designed with FLX titanium adapter sequences: A, adapter 5′-CCA TCT CAT CCC TGC GTG TCT CCG ACT CAG-3′, B, adapter 5′-CCT ATC CCC TGT GTG CCT TGG CAG TCT CAG-3′, and a barcode attached to the reverse primer. Forward primers contained the B adapter and the reverse primers contained the A adapter. Thus, the structure was forward primer-B adapter-barcode-reverse primer-A adapter. Bacterial amplicons were pyrosequenced on a 454 Genome Sequencer GS FLX Titanium platform (Roche Diagnostics) at Plataforma de Sequenciamento de Alto Desempenho—Fundação Oswaldo Cruz (FIOCRUZ, Rio de Janeiro.

### Pyrosequencing data processing and analysis

A detailed description of (i) data processing and (ii) analyses are provided in S1 Table. Briefly, raw data (176.299 sequences) were processed using the Quantitative Insights Into Microbial Ecology (QIIME) software package v. 1.9.1 [40] and Galaxy (https://main.g2.bx.psu.edu/; [41]. After quality check, removal of noise, chimera, chloroplast, unassigned (without taxonomy assignment at domain level) and singleton OTUs, the sequences were then trimmed to their maximum length and sorted according to the 8-mer barcodes. The filtered data set was used for downstream analyses. These initial steps were performed for both hypervariable regions (V1-V3 and V3-V5) sequenced. However, taken into consideration the number and the size of the sequences after quality control, only the data generated with the V1-V3 regions were further processed. After processing, 73.449 sequences were further analyzed within the QIIME environment to determine the Operational Taxonomic Units (OTUs) defined at ≥97% 16S rRNA gene sequence similarity and taxonomic assignment. This was followed by the generation of a table containing the OTUs per sample using customized scripts (S1 Table). Data analysis encompassed (i) estimates of the bacterial richness (Chao1) and diversity (Shannon diversity index), (ii) phylum- and class-level bacterial composition in individual and pooled samples, (iii) assessment of specific and shared bacterial OTUs across sample groups via OTU networks and Venn diagrams, and (iv) multivariate analysis of OTU data performed by Principal Coordinate Analysis (PCoA) of OTU profiles using the weighted Unifrac metric within QIIME. Analyses were performed using full-size and size-normalized, quality-filtered sample libraries, hereafter called ‘full’ and ‘normalized’ data sets, respectively. The raw data and sequencing sample information have been submitted to the NCBI’s Sequence Read Archive (SRA) database under the accession number SRP116689.

### Functional characterization of the bacteriome using PICRUSt

Based on the 16S rRNA gene sequences of the full-size data set, we assigned putative functions using the Phylogenetic Investigation of Communities by Reconstruction of Unobserved States (PICRUSt) version 1.1.0. It is a bioinformatics tool that predicts functional profiles based on a marker gene, such as 16S rRNA gene sequences [42]. PICRUSt uses an extended ancestral-state algorithm that estimates the functional gene content of prokaryotes for which no genome is yet available. It is performed through evolutionary modeling of the copy number of each gene family, based on the phylogenetic relationship of the strains with all Bacteria for which sequenced genomes are available. In this study, PICRUSt was applied to predict metagenome gene functional content of each sample using the KEGG (Kyoto Encyclopedia of Genes and Genomes) database, therefore focusing in a set of KEGG orthologs (KOs). In the KEGG database, KOs are groups of homologous sequences, from numerous organisms from which specific molecular functions have been assigned. KOs are hierarchically organized and arranged into biological pathways. Noteworthy, due to functional similarity, some KOs might be present in more than one pathway. For more details, see S9 Table.

### Tests of significance

Measurements of richness and diversity from 454-pyrosequencing were tested with a single factor analysis of variance (ANOVA, which evaluates whether or not the mean values obtained for all groups were equal). Whereas the statistical significance of the relative abundances of the most dominant bacterial phyla and classes detected in the normalized and non-normalized datasets were tested with a two-factor unbalanced fixed (Model I) ANOVA with multiple comparisons of means by Tukey’s test. The significance of the treatments in the PCoA in the normalized and non-normalized datasets was performed with a single factor ANOVA with 999 permutations. All the above-mentioned analyses were performed with the stat package in R programming (R Development Core Team).

## Results

### PCR-DGGE fingerprinting of bacterial communities

**Fig 1:**
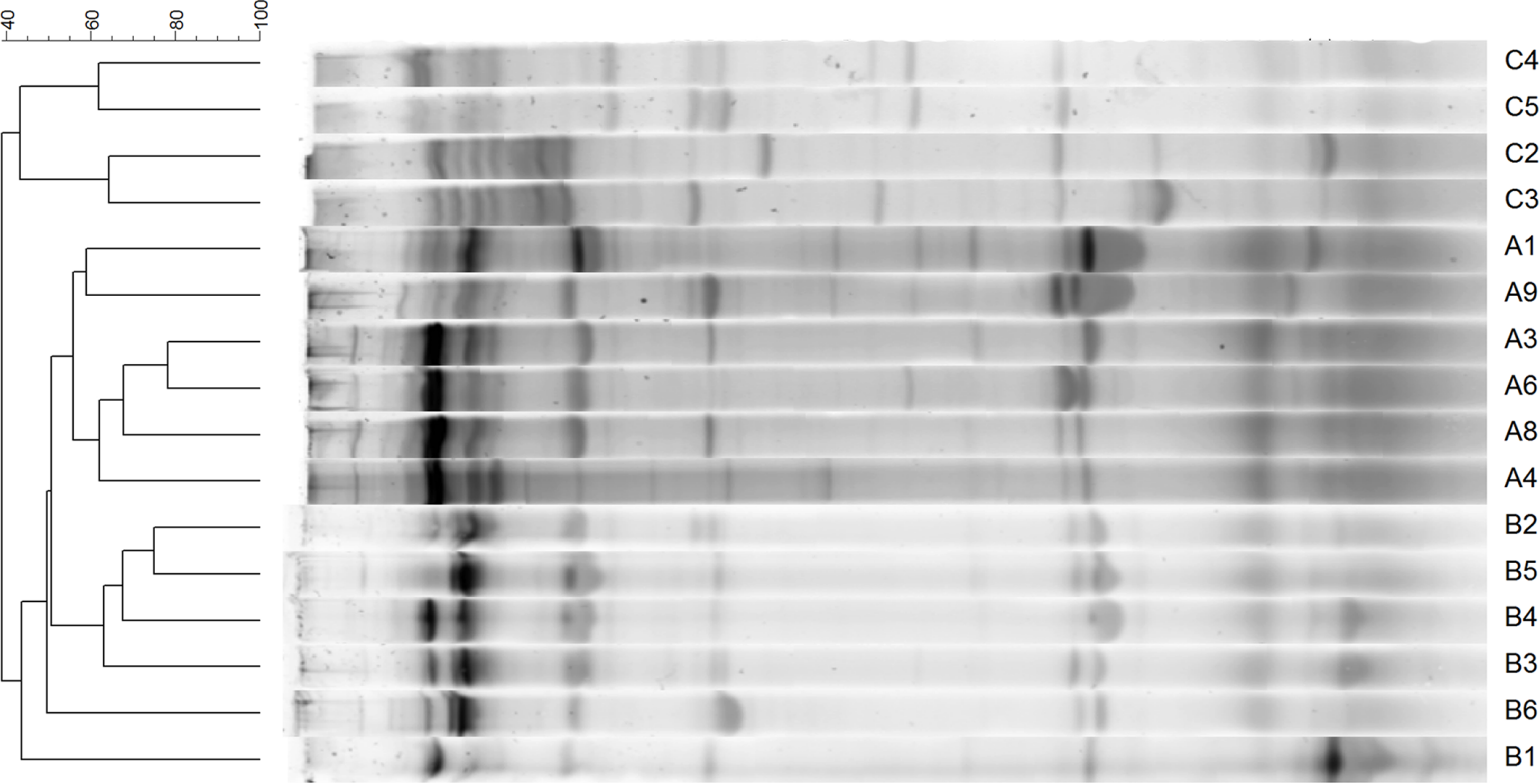
Dendrograms derived from DGGE analysis of the gut microbiota. UPGMA-type dendrograms were constructed based on the similarity matrix resulting from Pearson’s pair-wise comparisons of DGGE fingerprints. A- low violacein dose, B- high violacein dose, C- control group.

The 16S rRNA gene-based PCR-DGGE analyses revealed that the intake of violacein for a month induced changes in the composition of the gut microbiota when compared to the control samples without violacein. Fig 1 shows a clear clustering of patterns according to the treatment. In the control group (n = 4) the samples clustered together (i.e. 50% community profile similarity), whereas the low violacein (group A; n = 6) and high violacein groups (group B; n = 6) clustered apart from the control (i.e. less than 40% community profile similarity).

**Fig 2:**
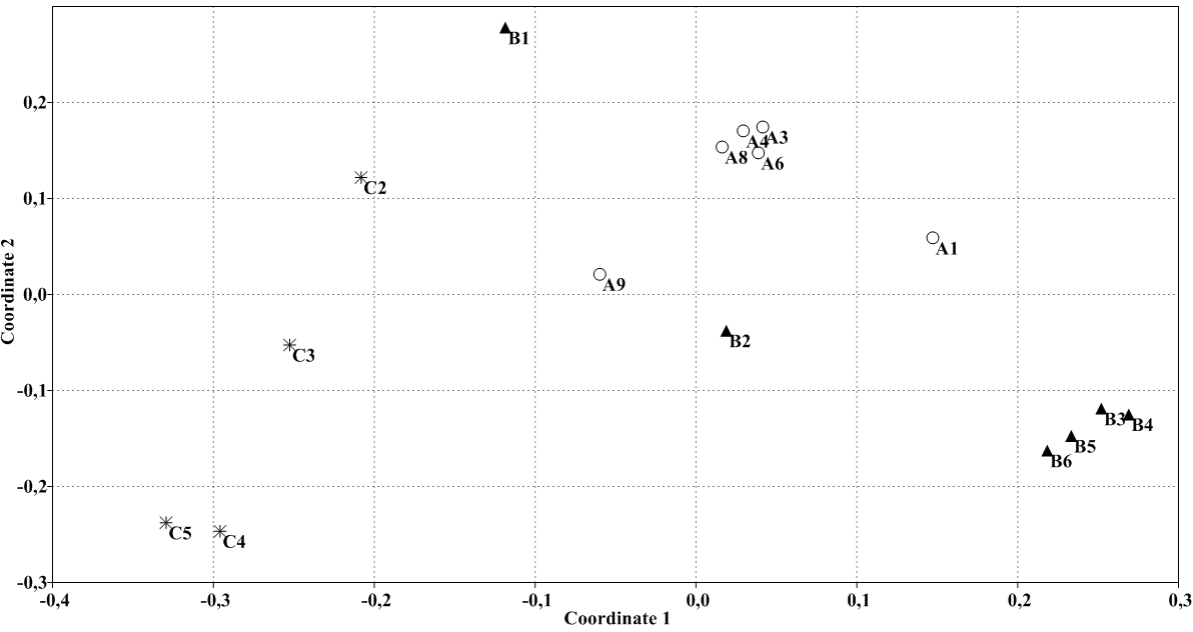
NMDS plot based on Bray-Curtis distances of the gut microbiota. Each data point represents one sample from the control (✳), low violacein (○) and high violacein (▲) groups.

Non-metric dimensional scaling (NMDS) of the data clearly indicated a dichotomy in the clustering (Fig 2). The patterns derived from the control group were closely related, as well as the patterns from the low violacein group. In contrast, the patterns of the high violacein group clustered apart. PERMANOVA confirmed the results of the NMDS analysis, demonstrating that the bacterial assemblages varied significantly among the control, low violacein and high violacein dose patterns (*p* = 0.0002). All these data confirm the differences between the three groups, although a more expressive similarity is highlighted in samples from the low violacein group.

### 454-pyrosequencing

After quality control, noise filtering with QIIME and trimming with Galaxy, 81153 16S rRNA V1-V3-tag sequences were further analyzed with QIIME. Then, chimera, chloroplast, unassigned and singleton sequences were removed from the OTU table, resulting in 73449 sequences that were assigned to 853 OTUs at 97% sequence similarity (S1 Table) in the full-size data set. The size-normalized libraries contained 56144 sequences (7018 sequence reads per sample), which were assigned to 835 OTUs at 97% sequence similarity. S2 Table shows the taxonomy affiliation as well as the number of sequences of each OTU for the size-normalized data set.

### Bacterial richness and diversity

**Fig 3:**
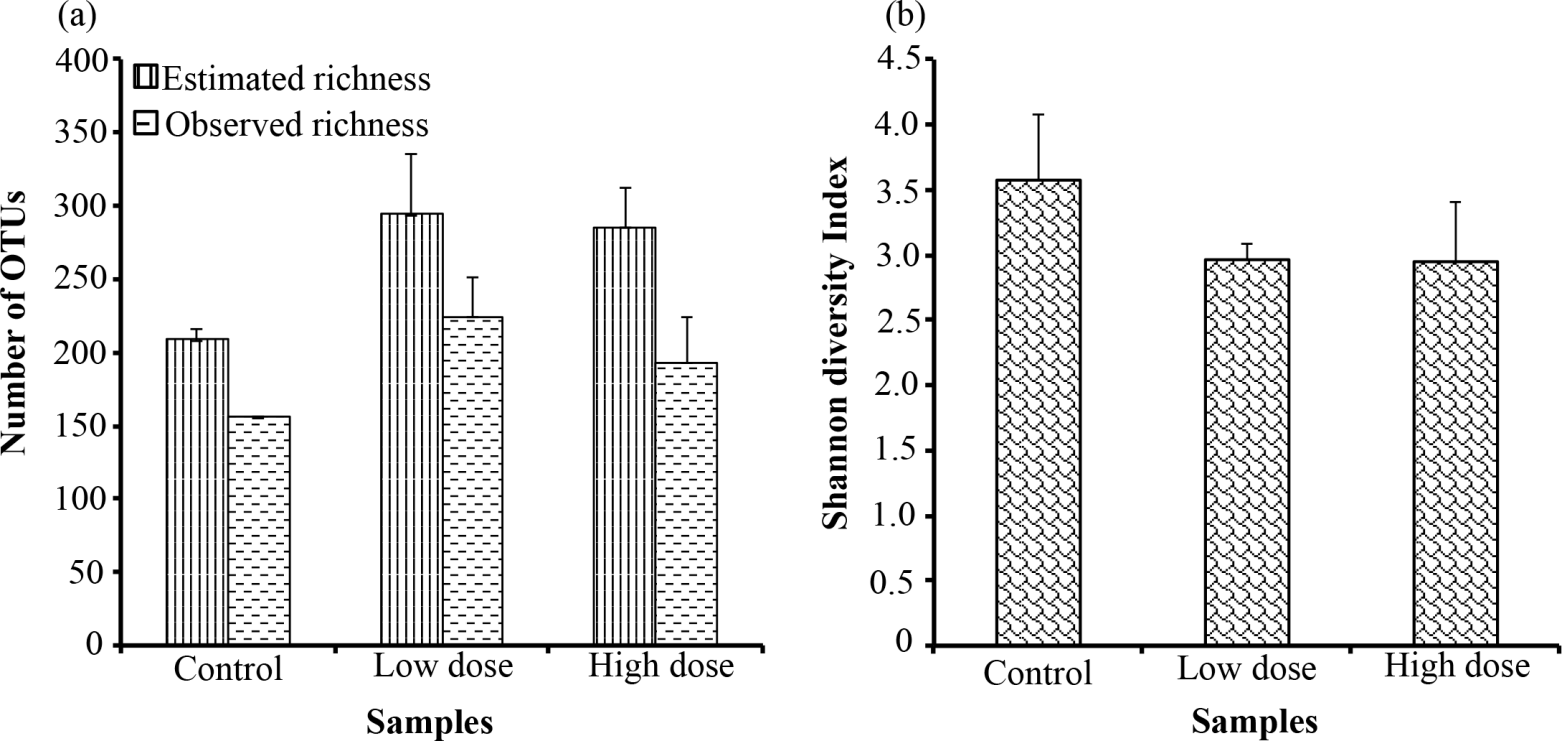
Bacterial richness and diversity. Observed and estimated (Chao1) richness measures (a) and Shannon diversity index (b) detected from control, low violacein dose and high violacein dose categories. Results were obtained from size-normalized data set (7018 bacterial sequence reads per sample).

To avoid biases related to the amount of sequences in each library, the results from richness and diversity measurements will be presented only for the size-normalized libraries. With equal numbers of sequences, the observed bacterial richness recovered from the control, low and high violacein groups were 156±0.05, 224.4±27.24 and 193.3±31.01, respectively (Fig 3A). The highest richness was observed for the low violacein group, followed by the high violacein group and then the control group. The Shannon diversity index was higher in the control samples (3.6±0.51) when compared to low (2.96±0.12) and high (2.94±0.46) violacein groups (Fig 3B). However, no significant difference (*p* > 0.05) was detected among the groups for richness and diversity measurements.

### Bacterial community composition

**Fig 4:**
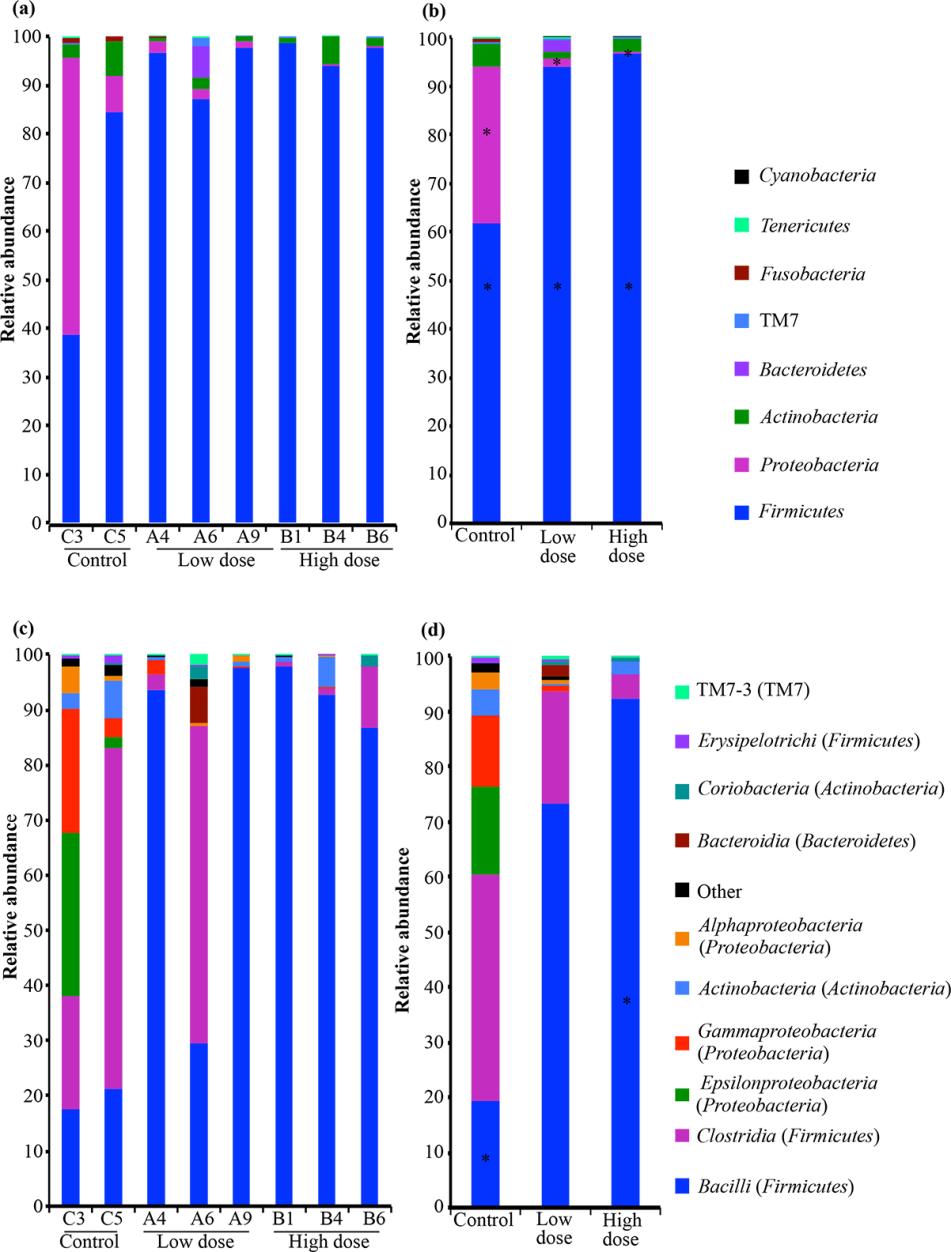
Composition of gut microbiota at phylum and class level. Phylum- (a, b) and class-level (c, d) bacterial community composition in the control, low and high violacein dose categories using size-normalized data set (7018 sequence reads per sample). The compositions of each replicate sample (a, c) and of pooled replicate samples (b, d) are presented. Asterisks on bars denote dominant taxa displaying significant shifts in relative abundance when control group was compared with low and high violacein dose treatments. An equivalent analysis including all 853 OTUs was performed with the full-size data set (S1 Fig).

The 16S rRNA gene sequences from size-normalized libraries were assigned to eight bacterial phyla (Actinobacteria, Bacteroidetes, Cyanobacteria, Firmicutes, Fusobacteria, Proteobacteria, Tenericutes, and TM7), but not all phyla were detected in all samples. Firmicutes was the most abundant phylum detected (86.9% average relative abundance), except for one replicate from the control, which was dominated by Proteobacteria (56.9% relative abundance) (Fig 4A and 4B). A total of 129 OTUs in 8657 sequences, 431 OTUs in 19768 sequences, and 370 OTUs in 20390 sequences were identified as Firmicutes for control, low and high violacein dose groups, respectively (Table 1). The second most abundant phylum was Proteobacteria, with 80 OTUs in 4531 sequences in the control, 17 OTUs in 409 sequences for the low violacein group and 10 OTUs in 36 sequences for the high violacein group (Table 1). Proteobacteria encompassed 8.86% of average relative abundance and was absent in one replicate from the high violacein group. The third most dominant phylum was Actinobacteria, present in all sample categories, with 2.77% average relative abundance. It was comprised of 24 OTUs in 677 sequences, 26 OTUs in 270 sequences, and 39 OTUs in 609 sequences for control, low and high violacein dose groups, respectively (Table 1). The bacterial phyla Bacteroidetes, Cyanobacteria, Fusobacteria, Tenericutes and TM7 accounted for 1.42% of the sequences (average relative abundance). Statistics analyses revealed that Firmicutes and Proteobacteria were significant different (*p* < 0.05) in the control group when compared with low and high violacein dose groups. Similar trends were observed when analyses were carried out with the full-size data set (Figures A and B in S1 Fig; S3 Table).

**Table 1:** Distribution of the number of OTUs and sequences for the bacterial phyla across all categories for the size-normalized data set.

	Control		Low dose		High dose	
	OTUs	Seqs	OTUs	Seqs	OTUs	Seqs
Actinobacteria	24	677	26	270	39	609
Bacteroidetes	2	3	31	465	0	0
Cyanobacteria	0	0	1	2	1	2
Firmicutes	129	8.657	431	19.768	370	20.390
Fusobacteria	1	133	2	3	0	0
Proteobacteria	80	4.531	17	409	10	36
Tenericutes	2	14	3	6	1	1
TM7	2	21	11	131	5	16
**Total**	**240**	**14.036**	**522**	**21.054**	**426**	**21.054**

Values correspond to quality-filtered OTUs and sequences across the size-normalized data set. Seqs = sequences

When lower taxonomic bacterial levels were assessed, 17 classes were identified. However, not all classes were registered in all samples. Bacilli and Clostridia largely dominated the sequences assigned to Firmicutes. Together, these classes accounted for 42.3% (average relative abundance) of the bacterial community composition across samples (Fig 4C and 4D). Bacilli dominated the pool of Firmicutes for all replicates of the high violacein dose group and two replicates from the low violacein dose group, with relative abundance ranging from 86.7 to 97.83% (Fig 4C and 4D). Bacilli comprised 60 OTUs in 2725 sequences, 318 OTUs in 15473 sequences and 319 OTUs in 19451 sequences for the control, low and high violacein dose groups, respectively (Table 2). The class Clostridia, also within the Firmicutes, was enriched in both control samples and one replicate from the low violacein group (Fig 4C). Its relative abundance ranged from 20.5 to 61.8% (Fig 4C and 4D). The class Clostridia encompassed 65 OTUs in 5780 sequences for the control category, 108 OTUs in 4268 sequences for the low violacein dose and 47 OTUs in 923 sequences for the high violacein dose (Table 2). For the control samples, the classes Actinobacteria and Epsilon- and Gamma-proteobacteria were also abundant, with 23 OTUs in 675 sequences, 22 OTUs in 2211 sequences and 42 OTUs in 1825 sequences, respectively (Table 2). Their average relative abundance were 15.75, 13 and 4.8%, respectively (Fig 4C and 4D). The classes where the sum across all samples was below 2% are shown as “others” in Fig 4C and 4D. They encompassed Beta- and Delta-proteobacteria (Proteobacteria), Fusobacteriia (Fusobacteria), Mollicutes (Tenericutes), Saprospirae (Bacteroidetes), unclassified class within Firmicutes, and 4C0d-2 (Cyanobacteria). Statistics analyses revealed that Bacilli (Firmicutes) was significant different (*p* < 0.05) in the control group compared with high violacein dose groups. When analyses were performed with full-size libraries, similar results were obtained, except for Fusobacteriia (Fusobacteria), which had an abundance superior to 2% (Figures C and D in S1 Fig, S4 Table).

**Table 2:** Distribution of the number of OTUs and sequences for the bacterial classes across all categories for the size-normalized data set.

	Control		Low dose		High dose	
	OTUs	Seqs	OTUs	Seqs	OTUs	Seqs
Actinobacteria (Actinobacteria)	23	675	11	101	21	462
Coriobacteriia (Actinobacteria)	1	2	15	169	18	147
Saprospirae (Bacteroidetes)	1	2	0	0	0	0
Bacteroidia (Bacteroidetes)	1	1	31	465	0	0
4C0d-2 (Cyanobacteria)	0	0	1	2	1	2
Unclassified (Firmicutes)	0	0	0	0	1	6
Bacilli (Firmicutes)	60	2.725	318	15.473	319	19.451
Clostridia (Firmicutes)	65	5.780	108	4.268	47	923
Erysipelotrichi (Firmicutes)	4	152	5	27	3	10
Fusobacteriia (Fusobacteria)	1	133	2	3	0	0
Alphaproteobacteria (Proteobacteria)	10	390	7	126	3	16
Betaproteobacteria (Proteobacteria)	6	105	2	11	0	0
Deltaproteobacteria (Proteobacteria)	0	0	1	100	1	2
Epsilonproteobacteria (Proteobacteria)	22	2.211	0	0	2	2
Gammaproteobacteria (Proteobacteria)	42	1.825	7	172	4	16
Mollicutes (Tenericutes)	2	14	3	6	1	1
TM7-3 (TM7)	2	21	11	131	5	16
**Total**	**240**	**14.036**	**522**	**21.054**	**426**	**21.054**

Values correspond to quality-filtered OTUs and sequences across the size-normalized data set. Seqs = sequences

### Specificities and commonalities of OTUs

**Fig 5:**
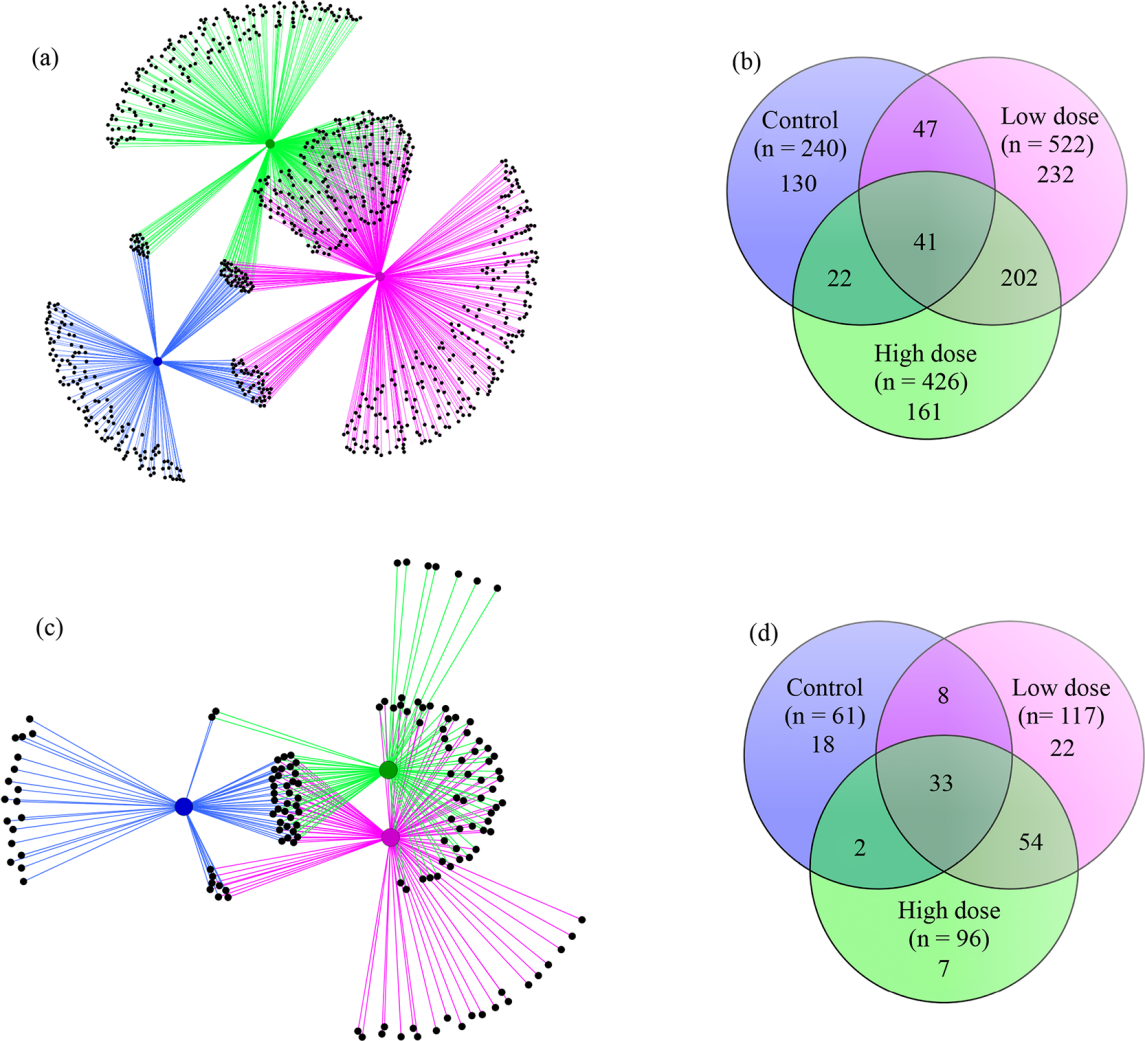
Specificities and commonalities of OTUs. A bacterial OTU network made with all 835 OTUs detected in composite samples where replicates were pooled according to the category (a). In the network, blue, magenta and green lines correspond to control, low and high violacein dose groups, respectively. Venn diagram showing the OTUs that were exclusive to each category or shared between or among categories (b). These analyses were carried out with size-normalized library sizes (7018 sequence reads per sample). Similar analyses were also performed for the full-size data set (S2 Fig).

An OTU network depicting the assignment of all 835 bacterial OTUs detected in this study to their sample categories was created (Fig 5A). The distribution of the categories in the network clearly demonstrated that the bacterial OTUs registered in the control group were distinct from those assigned in the low and high violacein dose categories. It also showed that the number of specific OTUs recorded in the control group was smaller when compared to the other two categories. Moreover, it seemed that about half of the OTUs detected in the low dose violacein group was specific to this category. Furthermore, there were more OTUs shared between the low and high violacein categories than between each of these categories and the control group (Fig 5A).

These trends were further investigated and quantified with Venn diagrams (Fig 5B). These analyses revealed that 130, 232 and 161 OTUs were specific to the control, low and high violacein dose categories, respectively. The Proteobacteria was the most abundant phyla exclusively associated with the control category, with 72 OTUs in 4230 sequences (Part A of S5 Table). Within it, the genus *Heliobacter* (class Epsilonproteobacteria) was dominant in terms of number of sequences (2123) and *Aggregatibacter pneumotropica* (Gammaproteobacteria) was dominant in number of OTUs (28) (Part A of S5 Table). For the low violacein dose group, the dominant phylum was Firmicutes, with 175 OTUs in 684 sequences; it was distributed in the classes Bacilli (117 OTUs in 418 sequences) and Clostridia (55 OTUs in 259 sequences) (Part B of S5 Table). Furthermore, the genus *Lactobacillus* (Bacilli) was the most abundant (69 OTUs in 275 sequences) (Part B of S5 Table). A similar trend was observed for the high violacein dose group, where Firmicutes was also the most abundant phylum (139 OTUs in 457 sequences) and the class Bacilli dominated its pool (117 OTUs in 380 sequences), with the genus *Lactobacillus* (83 OTUs in 282 sequences) contributing to its abundance (Part C of S5 Table). On the other hand, only 41 OTUs were common to all categories (the bacterial core), but not necessarily to all samples (Fig 5B). Among them, 29 OTUs were assigned to the phylum Firmicutes (44113 sequences), with the dominance of the class Bacilli (18 OTUs in 34891 sequences) (Part D of S5 Table). Notably, the genera *Lactobacillus* (5 OTUs in 23810 sequences) and *Streptococcus* (7 OTUs in 5652 sequences) contributed to the abundance of the class Bacilli (Part D of S5 Table). Remarkably, 202 OTUs were shared between the low and high violacein dose categories (Fig 5B). Firmicutes and Bacilli were the most abundant phylum (186 OTUs in 1912 sequences) and class (173 OTUs in 1678 sequences), respectively (Part E of S5 Table). In this class, the genus *Lactobacillus* (141 OTUs in 1224 sequences) dominated (Part E of S5 Table).

When analyses were performed with OTUs containing at least 10 sequences (in this scenario the “rare biosphere” was removed), the network revealed a drastic decrease in the overall number of OTUs (Fig 5C). The low violacein dose category had more specific OTUs in comparison with the two other categories (Fig 5C). There are more OTUs shared between the low and high violacein dose categories than OTUs common to: (i) control and low violacein dose categories, (ii) control and high violacein dose categories, and (iii) all categories (Fig 5C). The distribution of the categories within the network space demonstrated that the bacterial community was more similar between the low and high violacein dose categories than to the control category (Fig 5C).

These patterns were further confirmed with Venn diagram analyses. The total number of OTUs dropped from 835 to 144, from which 18, 22 and seven were exclusively detected in the control, low and high violacein dose categories, respectively (Fig 5D). Within this data set, the phylum Proteobacteria (11 OTUs in 3997 sequences) dominated the control category (Part A of S6 Table). The genus *Helicobacter* (Epsilonproteobacteria, two OTUs in 2107 sequences) and the species *Aggregatibacter pneumotropica* (Gammaproteobacteria, six OTUs in 1480 sequences) were the most abundant members of the Proteobacteria (Part A of S6 Table). For the low violacein dose category, the most abundant phylum in terms of number of OTUs was the Firmicutes (13 OTUs in 276 sequences), whereas in the number of sequences it was the Bacteroidetes (seven OTUs in 390 sequences) (Part B of S6 Table). For Firmicutes, the most common representatives were the genus *Lactobacillus* (Bacilli, seven OTUs in 120 sequences) and the order Clostridiales (Clostridia, three OTUs in 108 sequences) (Part B of S6 Table). The family S24-7 (Bacteroidia, seven OTUs in 390 sequences) dominated the pool of Bacteroidetes (Part B of S6 Table). For the high violacein dose category, the most abundant phylum was Firmicutes (five OTUs in 133 sequences) and the genus was *Lactobacillus* (Bacilli, three OTUs in 104 sequences) (Part C of S6 Table). The bacterial core encompassed 33 OTUs, and was dominated by the phylum Firmicutes (22 OTUs in 44080 sequences). The genus *Lactobacillus* (Bacilli, four OTUs in 23806 sequences), the family Clostridiaceae (Clostridia, three OTUs in 9107 sequences) and the genus *Streptococcus* (Bacilli, six OTUs in 5634 sequences) were the most representative components of the phylum Firmicutes (Part D of S6 Table). Fifty-four OTUs were common to the low and high violacein dose categories. Within it, the most abundant members were the phylum Firmicutes (46 OTUs in 1321 sequences) and the genus *Lactobacillus* (Bacilli, 35 OTUs in 769 sequences) (Part E of S6 Table). These analyses were also carried out with the full-size data set. Similar trends were also observed when all 853 OTUs as well as the 172 OTUs with a minimum of 10 sequences were used (S2 Fig, S7 and S8 Tables). The exception was that the control group showed more specific OTUs in comparison with the other two categories (Figure D in S2 Fig).

### Ordination of bacterial OTUs

**Fig 6:**
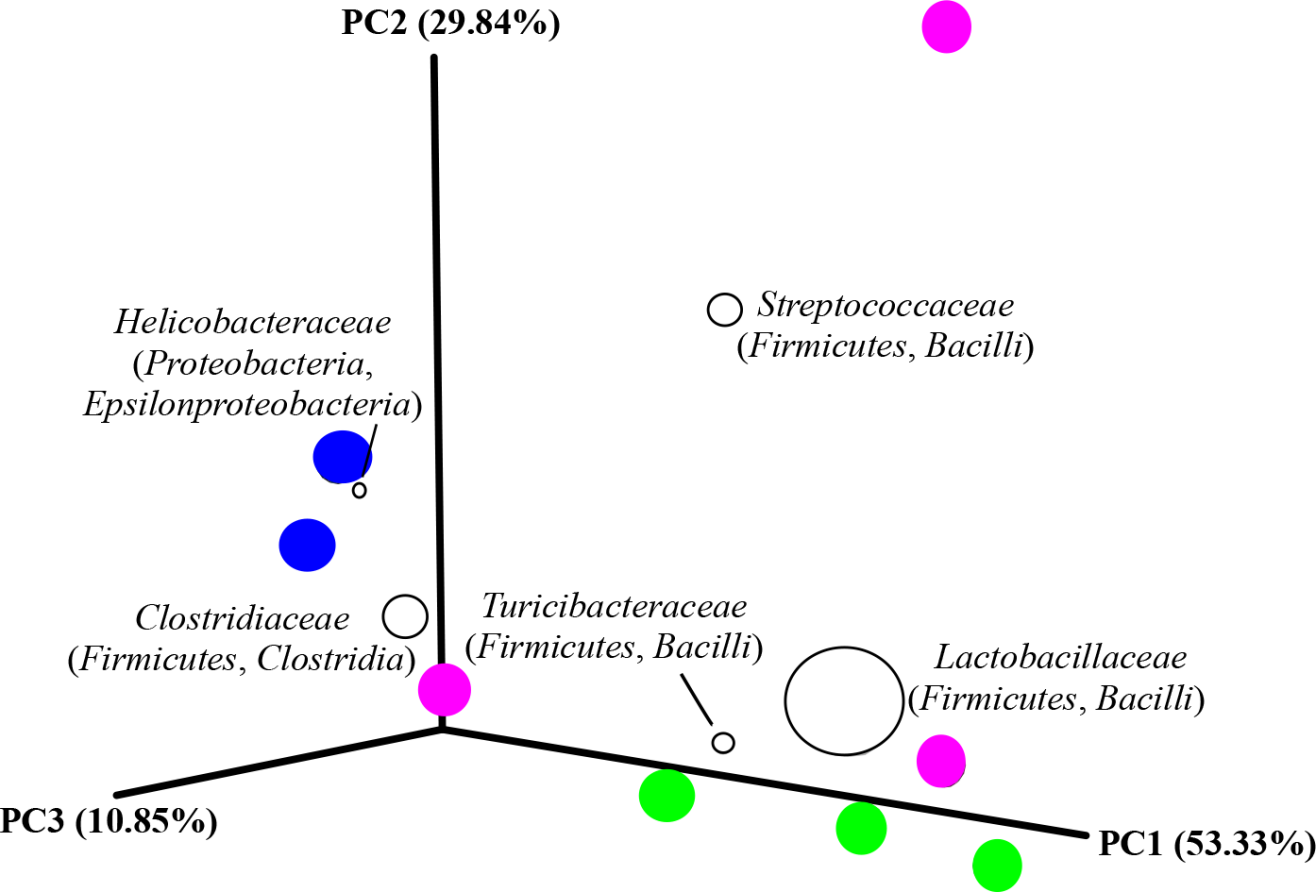
Ordination of bacterial OTUs. Principal coordinate analysis (PCoA) of all 835 OTUs using the weighted UniFrac metric for the size-normalized data set (7018 sequence reads per sample). Blue, magenta and green circles correspond to samples from control, low and high violacein dose groups, respectively. The five most dominant bacterial taxa (at family level) are presented. The size of their symbols represents the respective, mean relative abundance across the data set. The correlation between the bacterial taxa abundances and the treatment determined the position of them in the ordination space.

Principal coordinate analysis revealed that the replicates from the control grouped together and apart from the replicates from the other two categories. This seemed to be related to the dominance of the family Helicobacteraceae (Proteobacteria, Epsilonproteobacteria) in their bacterial community structure (Fig 6). On the other hand, the replicates from the low violacein dose group were scattered. Considering the distribution of the replicates from the low violacein dose in the PCoA, it appeared that each replicate had a distinct dominant Firmicutes family (Fig 6). The high violacein dose replicates grouped together, and this was most likely due to the abundance of the family Lactobacillaceae (Firmicutes) (Fig 6). The PCoA clearly demonstrated the dominance of the family Lactobacillaceae (Firmicutes) among the samples, especially for all replicates of the high violacein dose group and two replicates of the low violacein dose group (Fig 6). Statistic analysis demonstrated that there was not significant difference among the groups.

### PICRUSt

The assignment of putative functions based on the phylogenetic marker 16S rRNA gene of all samples allowed the identification of 5151 KEGG Orthologs (KOs) entries. For robustness of this analysis, we removed KO entries with less than 100 sequences across all samples. Thus, the analyses were performed with 3442 KOs entries (S9 Table). These KOs entries were further used for a gene set enrichment analysis to known KEGG pathways by comparing (i) control and low violacein dose groups, (ii) control and high violacein dose groups, and (iii) low and high violacein dose groups.

**Fig 7:**
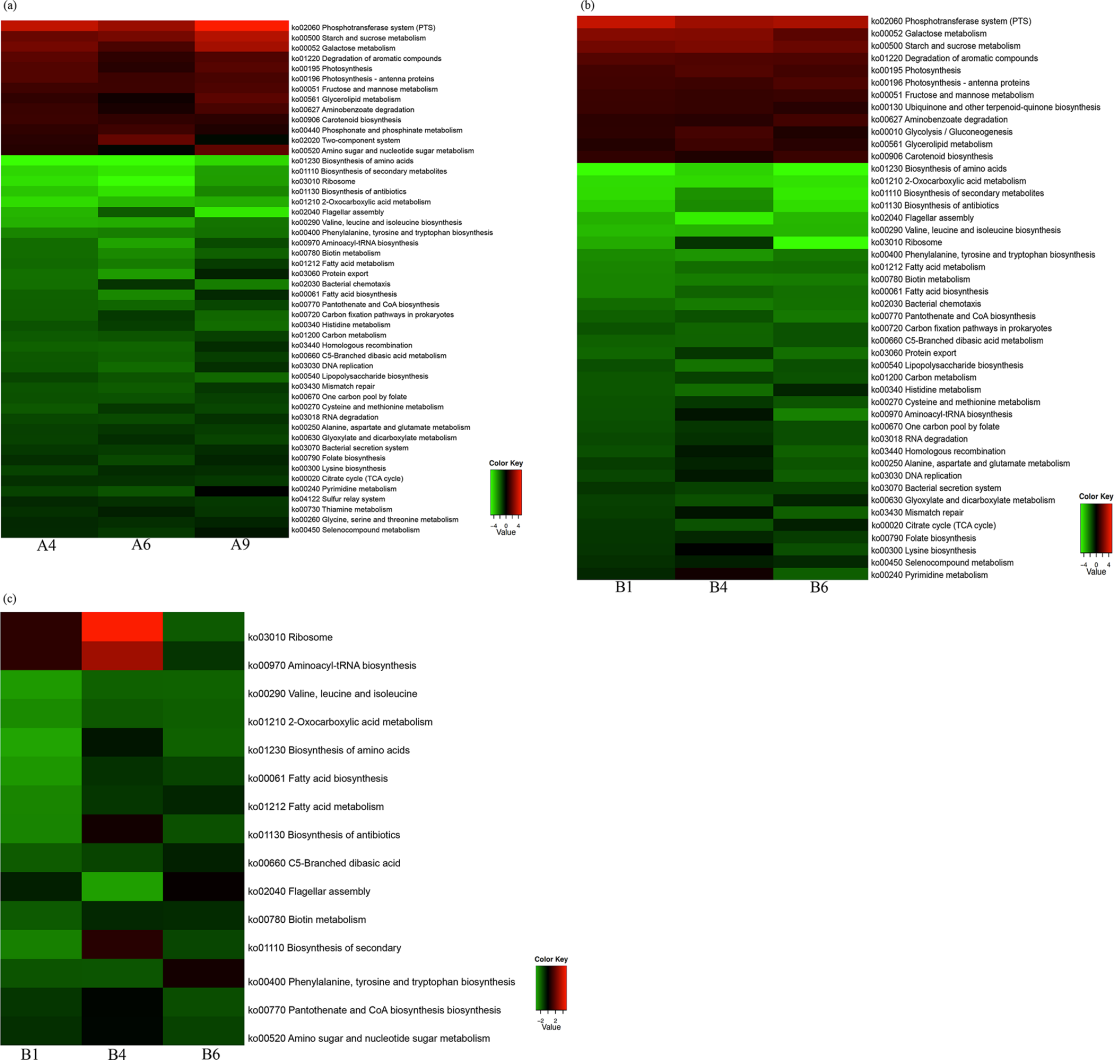
Analysis of KEGG pathways. Heatmap tables showing the enrichment analysis of KEGG pathways or KEEG ortholog entries for the comparison between: (a) low violacein dose and control, (b) high violacein dose and control, and (c) low and high violacein dose categories. In a and b, when functions in the pathway of treated samples were more abundant than in the control samples the pathways are shown in red colors, whereas pathways enriched in control samples are shown in green colors. In c, the functions enriched in the low violacein dose samples were shown in green colors, while the opposite is shown in red colors.

Regarding the first comparison (control *vs* low violacein dose), a total of 13 KEGG pathways or KO systems were more abundant in the low violacein dose. These entries include phosphotransferase system (PTS), starch and sucrose metabolism, galactose metabolism, degradation of aromatic compounds, photosynthesis, photosynthesis—antenna proteins, fructose and mannose metabolism, glycerolipid metabolism, aminobenzoate degradation, carotenoid biosynthesis, phosphonate and phosphinate metabolism, two-component system, and amino sugar and nucleotide sugar metabolism (Fig 7A). On the other hand, the number of KEGG pathways enriched in the control samples was higher, with 37 entries. These encompassed biosynthesis of amino acids, biosynthesis of secondary metabolites, ribosome, biosynthesis of antibiotics, 2-oxocarboxylic acid metabolism, flagellar assembly, valine, leucine and isoleucine biosynthesis, phenylalanine, tyrosine and tryptophan biosynthesis, aminoacyl-tRNA biosynthesis, biotin metabolism, fatty acid metabolism, protein export, bacterial chemotaxis, fatty acid biosynthesis, pantothenate and CoA biosynthesis, carbon fixation pathways in prokaryotes, histidine metabolism, carbon metabolism, homologous recombination, C5-branched dibasic acid metabolism, DNA replication, lipopolysaccharide biosynthesis, mismatch repair, one carbon pool by folate, cysteine and methionine metabolism, RNA degradation, alanine, aspartate and glutamate metabolism, glyoxylate and dicarboxylate metabolism, bacterial secretion system, folate biosynthesis, lysine biosynthesis, citrate cycle (TCA cycle), pyrimidine metabolism, sulfur relay system, thiamine metabolism, glycine, serine and threonine metabolism, and seleno-compound metabolism (Fig 7A).

There were 12 KEGG pathways enriched in the high violacein dose group when compared to the control: phosphotransferase system (PTS), galactose metabolism, starch and sucrose metabolism, degradation of aromatic compounds, photosynthesis, photosynthesis—antenna proteins, fructose and mannose metabolism, ubiquinone and other terpenoid-quinone biosynthesis, aminobenzoate degradation, glycolysis/gluconeogenesis, glycerolipid metabolism, and carotenoid biosynthesis (Fig 7B). The number of KEGG pathways enriched in the control was higher (34 entries) when compared to the high dose category. They resembled the ones abovementioned for the control category, except for the glycine, serine and threonine metabolism, thiamine metabolism, and sulfur relay system (Fig 7B).

The comparison between the low and high violacein dose groups revealed very few KEGG pathways enriched in the high violacein dose (ribosome and aminoacyl-tRNA biosynthesis) (Fig 7C), whereas 13 were more abundant in the low violacein dose: valine, leucine and isoleucine biosynthesis, 2-oxocarboxylic acid metabolism, biosynthesis of amino acids, fatty acid biosynthesis, fatty acid metabolism, biosynthesis of antibiotics, C5-branched dibasic acid, flagellar assembly, biotin metabolism, biosynthesis of secondary metabolites, phenylalanine, tyrosine and tryptophan biosynthesis, pantothenate and CoA biosynthesis, and amino sugar and nucleotide sugar metabolism (Fig 7C)

## Discussion

The composition of the gut microbiota is of great importance for the host and any modulation of this bacterial community may lead to a positive or negative effect on the host. Both the production of metabolites, such as short chain fatty acids, by the microbiota or direct interaction between this community and host cell can impact host health [43,44]. Treatment of bacterial infections is usually carried out with orally administered antibiotics, which will inevitably affect the microbiota living in the gut [45]. Many studies have reported the impact of different antibiotics on the gut microbiota. However, none of these was focused on violacein, a molecule that is known to exert various biological properties such as antibacterial activity [11].

As expected, administration of low and high doses of violacein for a month resulted in changes in the composition of the gut microbiota, as observed in DGGE and NMDS analyses. The shifts in community structure were distinct for the different doses of violacein, with close clustering of samples within treatment groups. Shifts in the bacterial communities and reduction of Shannon diversity in samples from animals treated with violacein were an expected result, since this pattern is commonly found in studies of antibiotic interference in the gut microbiota [46]. We observed that the highest number of species was detected in the treatment with a low dose of violacein, whereas the lowest richness was found in the control group. Treatments with violacein may be reducing the competitiveness of some bacterial populations such as proteobacteria leading to overgrowth of Bacilli (Firmicutes). Although these results point to marked changes in the gut microbial composition derived from violacein administration, we acknowledge that the reduced number of animals used in our study limits the conclusions that can be drawn from these experiments. Nevertheless, in many cases the effects observed with both treatments (low and high violacein dose) were similar, further supporting the notion that violacein treatment was indeed responsible for the phenotypes described herein.

The purity grade of the extracted violacein used in our study was not determined, so we cannot rule out the presence of biosynthetic intermediates and other organic compound contaminants, which could have unforeseen effects on the microbiome analysis. Nonetheless, unpublished results from Davi Barbirato have shown that extracted violacein and commercially available violacein (SIGMA; cat. V9389) have similar activity. Despite the proven role of violacein against some species in vitro, the mechanism of action behind this activity is still unknown. Some studies have shown that violacein can have synergistic activity with other compounds, like antimicrobials, potentializing their activities [47]. Further studies are needed to check if violacein is affecting the activity of bioactive small molecules produced in the gut, which would explain the discrepancy observed. The effect of violacein against Firmicutes is indeed documented, but it is important to point out that the phyla Firmicutes is composed of hundreds of bacterial genus and thousands of species. Although the overall effect on the Phyla is a reduction, some species within this group may be resistant to violacein and therefore increase in numbers due to violacein treatment.

Sequence analysis showed that all treatment groups had Firmicutes; however, when animals were treated with violacein, the rate of Firmicutes increased and the rate of Proteobacteria decreased. At the class level, we could notice alterations in the treatment groups, although all of them presented Bacilli and Clostridia (Firmicutes) as predominant. Other prevalent classes in the control group were Gammaproteobacteria (associated with pathogenic microorganisms and hepatic diseases)([48]) and Epsilonproteobacteria (Proteobacteria). Considering that the Proteobacteria phylum is composed of Gram-negative bacteria, reducing its members by violacein may exert an important role to diminish inflammation caused by these microorganisms. Besides Bacilli and Clostridia, other dominant class in the low violacein dose was Bacteroidia (Bacteroidetes), and in the high violacein dose were Actinobacteria and Coriobacteria (Actinobacteria). The gastrointestinal tract acts as a barrier, both physically and biochemically, against foreign antigens and pathogenic bacteria. Colonization by members of the gut microbiota, such as Bacteroidia, is responsible for the development of these protective functions by stimulating, for example, the expression of molecules with antibacterial activity or responsible for intercellular adhesion. Colonization can also suppress inflammation by modulating transduction of inflammatory signals or by inducing regulatory T cells [49,50]. Therefore, although violacein treatment altered the microbiota, this alteration could be beneficial for the host, since some Bacteroidias have been described as the most numerous and versatile polysaccharide users in the colon and have been shown to degrade a variety of plant oligo- and polysaccharides into short-chain fatty acids (SCFAs) [51]. Clinical evidence has linked the reduced number of Bacteroidetes in the microbiota to the unregulated immune responses observed in diseases such as Crohn's disease, ulcerative colitis, and celiac disease [52]. In this circumstance, the effect of the low violacein dose in increasing the amounts of Bacteroidia would be beneficial to the host. In the groups treated with violacein it was observed that the Lactobacillaceae family was increased when compared to the control. This family includes members that are important to promote health in the gut microbiota and great producers of lactic acid as a product from carbohydrate metabolism [53]. In the control group, the abundant family was Helicobacteraceae, which was reported to be associated to enteric diseases [53,54].

In order to evaluate which functions are enriched in the low and high violacein doses, we predicted metagenome gene functional content of each sample using the KEGG. Through this analysis, we observed the most noticeable changes in low violacein doses, with genes being enriched in functions that are often associated to carbohydrate metabolism such as starch and sucrose metabolism, fructose and mannose metabolism, as well as galactose metabolism, which are important in the initial steps in breaking down otherwise indigestible dietary polysaccharides. In conclusion, the present study shows that violacein treatment changes the composition of the mammalian gut microbiota. However, further investigation is required to identify how violacein would affect the microbiota in syndromes associated with the microbiota and whether this alteration would bring benefits to the host.

## Supporting information

S1 Protocol Human Microbiome Project (HMP) 454 16S Protocol Version 4.2.2. (PDF)

S1 Fig Composition of gut microbiota at phylum and class level. Phylum- (a, b) and class-level (c, d) bacterial community composition in the control, low violacein dose and high violacein dose categories using full-size libraries. The compositions of each replicate sample (a, c) and of pooled replicate samples (b, d) are presented. Asterisks on bars denote dominant taxa displaying significant shifts in relative abundance when control group was compared with low and high violacein dose treatments.
(TIF)

S2 Fig Specificities and commonalities of OTUs. A bacterial OTU network made with all 853 OTUs detected in composite samples where replicates were pooled according to the category (a). In the network, blue, magenta and red lines correspond to control, low and high violacein dose groups, respectively. Venn diagram showing the OTUs that were exclusive to each category or shared between or among categories (b). These analyses were carried out with the full-size libraries.
(TIF)

S1 Table 454-pyrosequencing data set summary. (DOCX)

S2 Table OTU table with the number of sequences per sample and their correspondent taxonomy affiliation. (XLSX)

S3 Table Distribution of the number of OTUs and sequences for the bacterial phylum across all categories for the full-size data set. (DOCX)

S4 Table Distribution of the number of OTUs and sequences for the bacterial class across all categories for the full-size data set. (DOCX)

S5 Table **Taxonomic classification and absolute abundance of the 835 bacterial OTUs found exclusively in the categories** (a) control, (b) low violacein dose, (c) high violacein dose, or shared (d) among all categories (the bacterial core) and (e) between low and high violacein doses for size-normalized data set.
(XLSX)

S6 Table **Taxonomic classification and absolute abundance of the 144 bacterial OTUs detected exclusively in the categories** (a) control, (b) low violacein dose, (c) high violacein dose, or shared (d) among all categories (the bacterial core) and (e) between low and high violacein doses for size-normalized data set.
(XLSX)

S7 Table **Taxonomic classification and absolute abundance of the 853 bacterial OTUs found exclusively in the categories** (a) control, (b) low violacein dose, (c) high violacein dose, or shared (d) among all categories (the bacterial core) and (e) between low and high violacein doses for full-size data set.
(XLSX)

S8 Table **Taxonomic classification and absolute abundance of the 172 bacterial OTUs detected exclusively in the categories** (a) control, (b) low violacein dose, (c) high violacein dose, or shared (d) among all categories (the bacterial core) and (e) between low and high violacein doses for full-size data set.
(XLSX)

S9 Table Number of sequences in each KEGG ortholog entry per sample. (XLSX)
