# Supplementary figures and images for: Impact of violacein from Chromobacterium violaceum on the mammalian gut microbiome
Source: PLoS One. 2018 Sep 13;13(9):e0203748. doi: 10.1371/journal.pone.0203748 (PMC6136722; doi:10.1371/journal.pone.0203748)

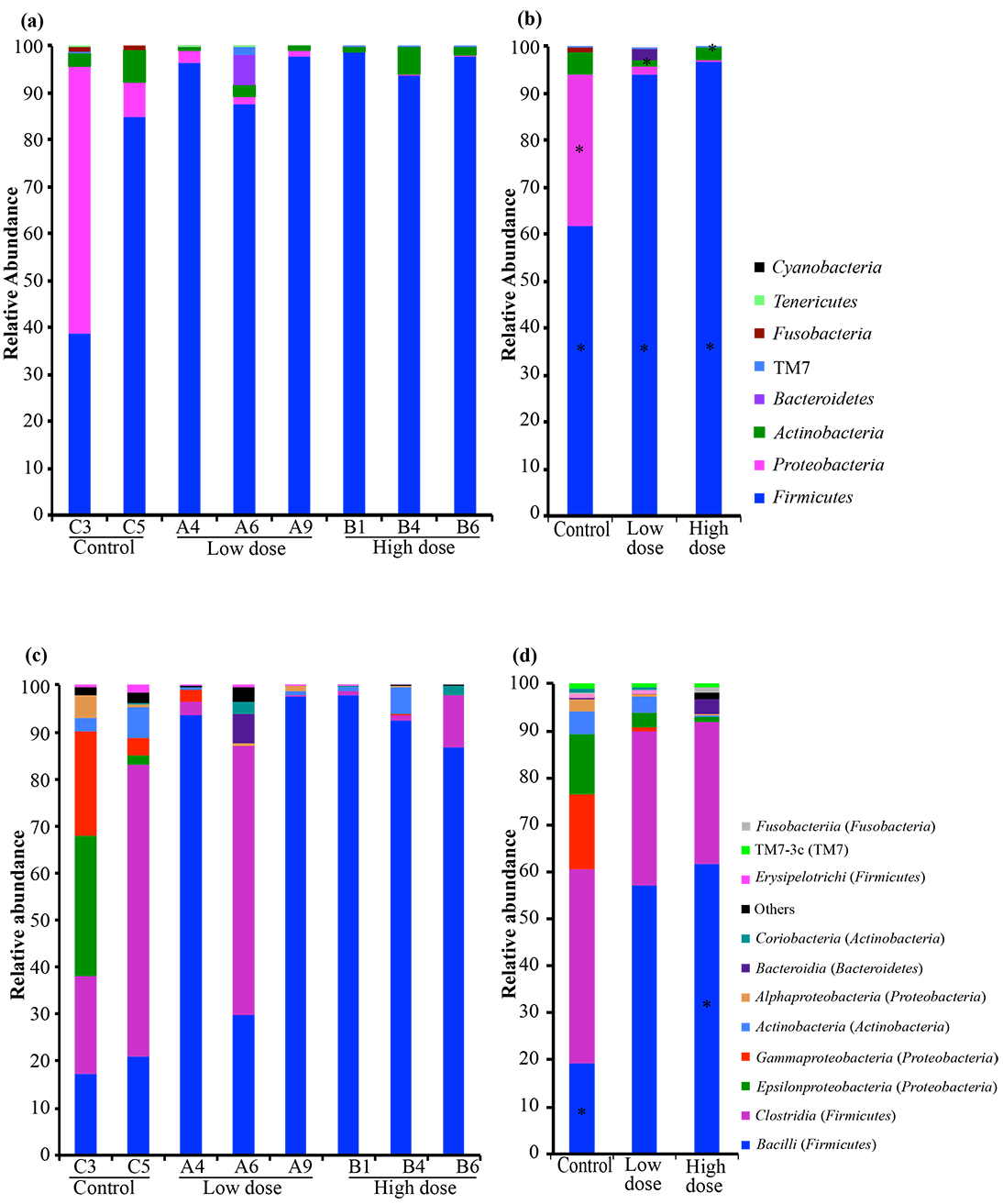

Supplement: S1 Fig — Phylum- (a, b) and class-level (c, d) bacterial community composition in the control, low violacein dose and high violacein dose categories using full-size libraries. The compositions of each replicate sample (a, c) and of pooled replicate samples (b, d) are presented. Asterisks on bars denote dominant taxa displaying significant shifts in relative abundance when control group was compared with low and high violacein dose treatments. (TIF) [file pone.0203748.s002.tif]

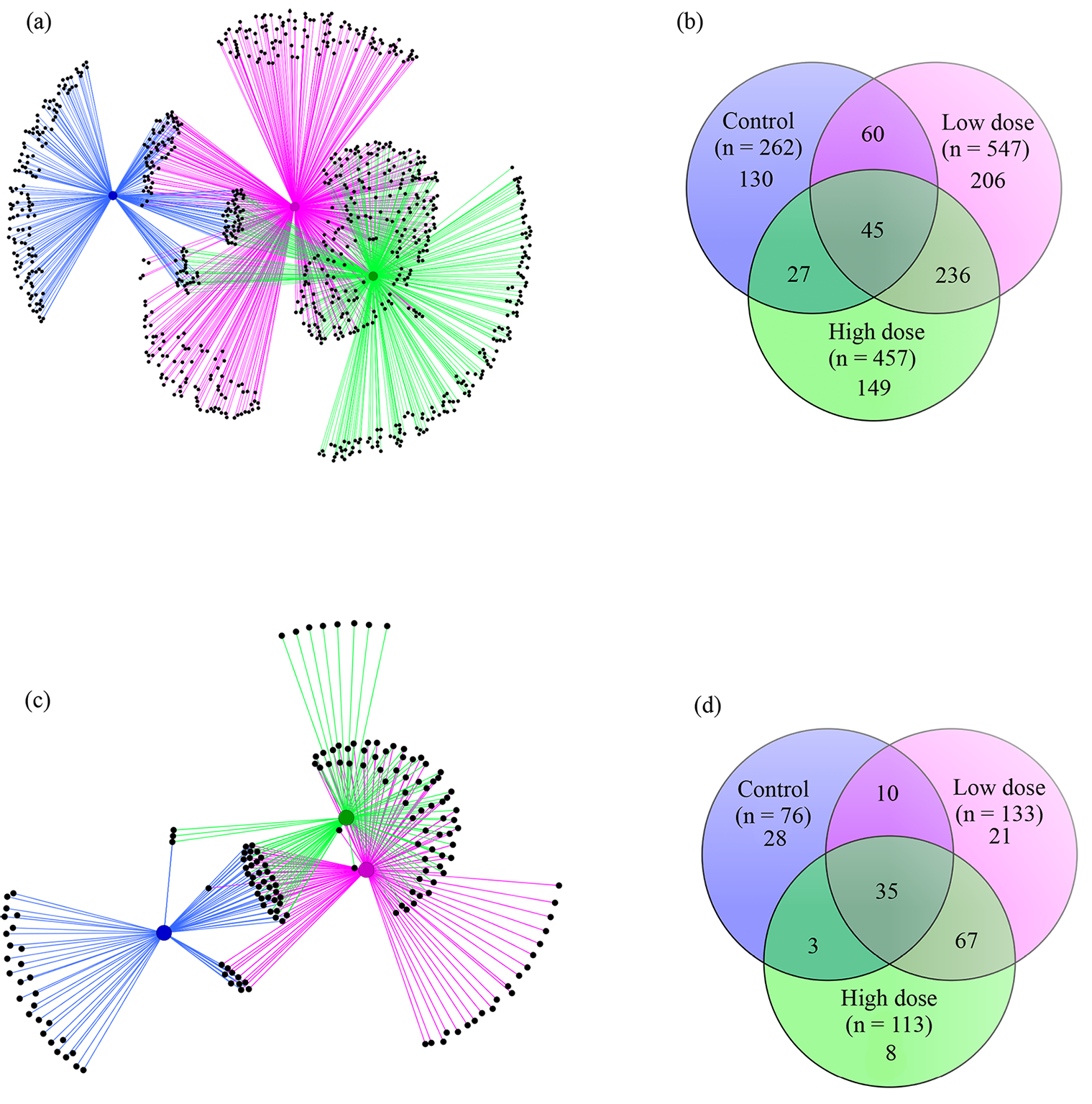

Supplement: S2 Fig — A bacterial OTU network made with all 853 OTUs detected in composite samples where replicates were pooled according to the category (a). In the network, blue, magenta and red lines correspond to control, low and high violacein dose groups, respectively. Venn diagram showing the OTUs that were exclusive to each category or shared between or among categories (b). These analyses were carried out with the full-size libraries. (TIF) [file pone.0203748.s003.tif]
