# Supplementary material for: Impact of violacein from Chromobacterium violaceum on the mammalian gut microbiome
Source: PLoS One. 2018 Sep 13;13(9):e0203748. doi: 10.1371/journal.pone.0203748 (PMC6136722; doi:10.1371/journal.pone.0203748)
Supplement: S1 Table — (DOCX) [file pone.0203748.s004.docx]

**Table S1.** 454-pyrosequencing data set summary.

|  |  | Before chimera removal and filtering OTU table | | | After chimera removal  and filtering OTU table | |
| --- | --- | --- | --- | --- | --- | --- |
| Sample | **Treatment** | | **OTUs 97%** | **Seqs** | **OTUs 97%** | **Seqs** |
| C3 | Control | | 444 | 13.374 | 188 | 12.452 |
| C5 | Control | | 279 | 9.672 | 171 | 9.272 |
| A4 | Low violacein dose | | 643 | 9.615 | 272 | 8.400 |
| A6 | Low violacein dose | | 779 | 9.172 | 241 | 7.674 |
| A9 | Low violacein dose | | 483 | 10.018 | 207 | 8.919 |
| B1 | High violacein dose | | 343 | 11.613 | 213 | 8.630 |
| B4 | High violacein dose | | 429 | 9.533 | 193 | 10.771 |
| B6 | High violacein dose | | 503 | 8.156 | 234 | 7.331 |
| Total | **n.a.** | | **2.768** | **81.153** | **853** | **73.449** |

n.a., not applicable.

*Seqs = sequences*
