# Supplementary material for: Impact of violacein from Chromobacterium violaceum on the mammalian gut microbiome
Source: PLoS One. 2018 Sep 13;13(9):e0203748. doi: 10.1371/journal.pone.0203748 (PMC6136722; doi:10.1371/journal.pone.0203748)
Supplement: S3 Table — (DOCX) [file pone.0203748.s006.docx]

|  |  |  |  |  |  |  |  |
| --- | --- | --- | --- | --- | --- | --- | --- |
| **Table S3.** Distribution of the number of OTUs and sequences for the bacterial phylum across all categories for the full-size data set. | | | | | | |  |
|  | **Control** | | **Low dose** | | **High dose** | | |
|  | **OTUs** | **Seqs** | **OTUs** | **Seqs** | **OTUs** | **Seqs** | |
| Actinobacteria | 27 | 968 | 26 | 304 | 41 | 863 | |
| Bacteroidetes | 2 | 7 | 31 | 505 | 0 | 0 | |
| Cyanobacteria | 0 | 0 | 1 | 2 | 1 | 2 | |
| Firmicutes | 146 | 12.669 | 454 | 23.538 | 396 | 25.782 | |
| Fusobacteria | 1 | 222 | 2 | 3 | 0 | 0 | |
| Proteobacteria | 82 | 7.801 | 18 | 486 | 12 | 64 | |
| Tenericutes | 2 | 25 | 3 | 11 | 1 | 1 | |
| TM7 | 2 | 32 | 12 | 144 | 6 | 20 | |
| **Total** | **262** | **21.724** | **547** | **24.993** | **457** | **26.732** | |

*Values correspond to quality-filtered OTUs and sequences across the full-size data set.*

*Seqs = sequences*
