# Supplementary material for: Impact of violacein from Chromobacterium violaceum on the mammalian gut microbiome
Source: PLoS One. 2018 Sep 13;13(9):e0203748. doi: 10.1371/journal.pone.0203748 (PMC6136722; doi:10.1371/journal.pone.0203748)
Supplement: S4 Table — (DOCX) [file pone.0203748.s007.docx]

**Table S4.** Distribution of the number of OTUs and sequences for the bacterial class across all categories for the full-size data set.

|  | **Control** | | **Low dose** | | **High dose** | |
| --- | --- | --- | --- | --- | --- | --- |
|  | **OTUs** | **Seqs** | **OTUs** | **Seqs** | **OTUs** | **Seqs** |
| Actinobacteria (Actinobacteria) | 26 | 965 | 11 | 117 | 22 | 699 |
| Coriobacteriia (Actinobacteria) | 1 | 3 | 15 | 187 | 19 | 164 |
| Saprospirae (Bacteroidetes) | 1 | 3 | 0 | 0 | 0 | 0 |
| Bacteroidia (Bacteroidetes) | 1 | 4 | 31 | 505 | 0 | 0 |
| 4C0d-2 (Cyanobacteria) | 0 | 0 | 1 | 2 | 1 | 2 |
| Unknown class (Firmicutes) | 0 | 0 | 0 | 0 | 1 | 6 |
| Bacilli (Firmicutes) | 65 | 4.102 | 337 | 18.839 | 341 | 24.740 |
| Clostridia (Firmicutes) | 77 | 8.347 | 110 | 4.666 | 50 | 1.020 |
| Erysipelotrichi (Firmicutes) | 4 | 220 | 7 | 33 | 4 | 16 |
| Fusobacteria (Fusobacteria) | 1 | 222 | 2 | 3 | 0 | 0 |
| Alphaproteobacteria (Proteobacteria) | 10 | 669 | 8 | 151 | 3 | 25 |
| Betaproteobacteria (Proteobacteria) | 6 | 151 | 2 | 12 | 1 | 1 |
| Deltaproteobacteria (Proteobacteria) | 0 | 0 | 0 | 0 | 1 | 2 |
| Epsilonproteobacteria (Proteobacteria) | 23 | 3.895 | 1 | 107 | 2 | 4 |
| Gammaproteobacteria (Proteobacteria) | 43 | 3.086 | 7 | 216 | 5 | 32 |
| Mollicutes (Tenericutes) | 2 | 25 | 3 | 11 | 1 | 1 |
| TM7-3 (TM7) | 2 | 32 | 12 | 144 | 6 | 20 |
| **Total** | **262** | **21.724** | **547** | **24.993** | **457** | **26.732** |

*Values correspond to quality-filtered OTUs and sequences across the full-size data set.*

Seqs = sequences
